# The Average Electron Density Tool for Bioisosterism in Hydrophobic Media

**DOI:** 10.1021/acsomega.5c04043

**Published:** 2025-08-05

**Authors:** Alya A. Arabi

**Affiliations:** College of Medicine and Health Sciences, Department of Biochemistry and Molecular Biology, United Arab Emirates University, AlAin, P.O. Box: 15551, United Arab Emirates

## Abstract

Solvent effects influence the electronic properties of drug-like molecules, including their behavior in bioisosteric design. This study investigates the impact of hydrophobic environments, modeled using the solvation model based on density (SMD), on the average electron density (AED) values of 63 bioisosteric moieties of carboxylic acid. Dipole moments and AED values were computed at the B3LYP-D3­(BJ)/6-311++G­(d,p) level of theory, followed by atom in molecule analysis. The AED values remained consistent, irrespective of the dielectric constant of the hydrophobic medium. These findings support the applicability of the AED tool for evaluating bioisosterism even in nonpolar environments such as the interior of a protein. Furthermore, solvent-induced changes in AED were evaluated under polar and nonpolar solvation, using the SMD model versus the integral equation formalism polarizable continuum model (IEFPCM). Results show that both models yield comparable AED values and, thus, either is suitable for studying AED while accounting for solvent effects.

## Graphical abstract

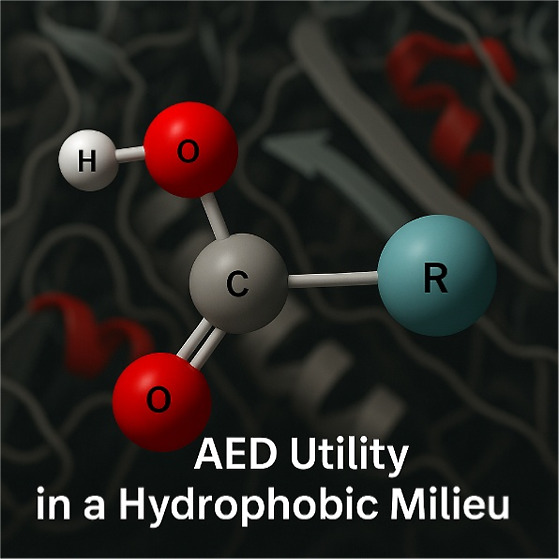

## Introduction

Bioisosterism is commonly used in rational drug design. Medicinal chemists use it to improve the pharmacokinetic and pharmacodynamic profiles of lead compounds by substituting a group of the molecule with different moieties, known as bioisosteres. This substitution fine-tunes the lead compound while preserving, or even enhancing, its biological activity. Bioisosteric replacements have been widely adopted to modulate drug potency, selectivity, absorption, distribution, metabolism, and excretion.^1–6^ These replacements can also improve solubility, permeability, metabolic stability, and reduce toxicity, all of which are critical for optimizing the therapeutic efficacy and safety profile of a given compound.^1–6^ Bioisosteres are classified into classical and nonclassical groups. Classical bioisosteres share the same number of valence electrons. Nonclassical bioisosteres are groups that differ significantly from one another in many properties, but have been reported to share similar AED values, specifically for bioisosteric moieties of carboxylic acid or amide.^7–12^ The AED tool can quantify the differences between nonclassical bioisosteric groups. The AED differences of nonclassical bioisosteric moieties, with respect to the carboxylic acid group, can be as small as 2%^9^ or even 0.15%,^10^ while the upper threshold of AED differences for experimentally tested nonclassical bioisosteres of carboxylic acid could reach 35%.^14^ The AED tool is applicable for neutral and anionic molecules, irrespective of the diversity in the capping groups.^7,11,12^ The AED tool has been also shown to accurately cluster conformers of a molecule according to the shape of their electrostatic potential maps, with accuracies exceeding 96%, as well as to match clusters of conformers of different molecules in such a way that they share similar electrostatic potential maps, *i.e.* similar interactions with a given receptor.^15^ For nonclassical bioisosteres of the amide group, AED values of methyl-capped moieties differed by no more than 4%.^12^ Overall, the published studies indicate that the AED tool is a reliable quantitative tool for evaluating nonclassical bioisosterism across diverse molecular systems and conditions, with performance that compares favorably to traditional qualitative approaches.

The AED tool has been tested under vacuum and under implicit aqueous solvation using IEFPCM, which is suitable in mimicking a polar hydrated environment.^16^ However, many drug molecules are found in nonaqueous microenvironments, such as the hydrophobic core of lipid bilayers or the interior of protein binding pockets, where electrostatic interactions and solvation dynamics differ from those in bulk water. The IEFPCM, while effective for simulating aqueous environments, is not built to adequately represent low-dielectric, nonpolar media. To address this limitation, this study explores the use of the SMD solvation model^17^ with the AED tool in assessing the nonclassical bioisosteres of the carboxylic acid moiety in media with low-dielectric constants.

In this study, the applicability of the AED tool in lipophilic or weakly polar milieus is investigated by using the SMD solvation model. This work advances the use of AED as a predictive tool in nonpolar biological contexts and offers insights into designing ligands for hydrophobic target sites. In addition, the SMD model will be used to evaluate the effect of aqueous solvation, with results to be compared to those from the IEFPCM model.^14^ This offers an insight about the performance of different solvation models with respect to AED in bioisosteric design in aqueous environments.

## Computational Methods

To explore the AED of nonclassical carboxylic acid bioisosteres under hydrophobic conditions, quantum chemical calculations were performed on a data set of 52 experimentally validated moieties obtained from ref 14 (see Figure 1). For chiral structures, both R and S enantiomers were considered (chiral centers are denoted by an asterisk in Figure 1). The input geometries of the methyl-capped bioisosteres were obtained from ref 14 and optimized at the B3LYP-D3­(BJ)/6-311++G­(d,p)//B3LYP-D3­(BJ)/6-311++G­(d,p) level, using the Gaussian16 package.^18^ This method includes dispersion which could be important in larger molecules in case intramolecular weak van der Waals interactions existed. Because conformers matter, and to minimize their effects, the optimized geometries were used as the starting point to reobtain the wave functions at the gas phase, as well as two relatively nonpolar media [characterized by dielectric constants of 2.04 (mimicked by *n*-hexadecane) and 6.25 (mimicked by acetic acid)], modeled using the SDM implicit solvation model.^18^ In biological environments, such low dielectric constants can be encountered inside proteins^19,20^ and in phospholipid bilayers.^21^

**Figure 1:**
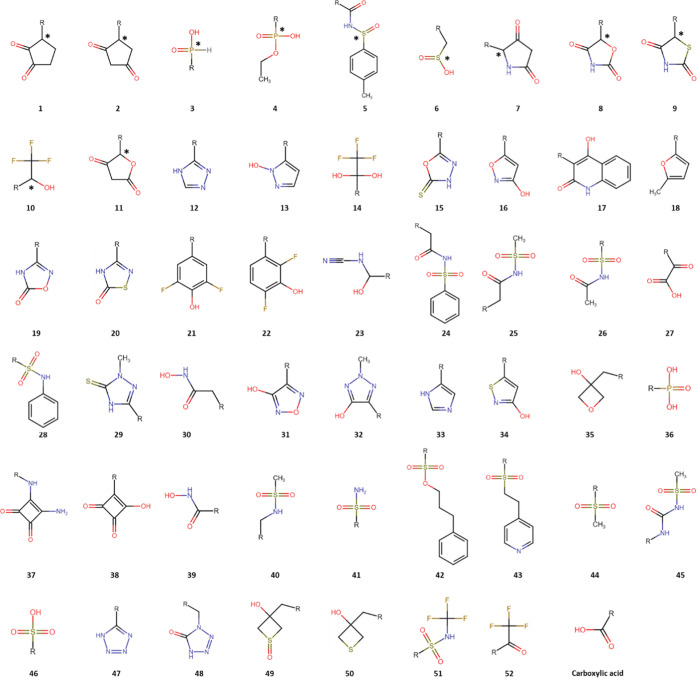
2D structures of the bioisosteric moieties considered in this study, each of which has a methyl capping group, denoted by an R. The asterisk denotes the chiral center in the 11 chiral bioisosteric moieties.

In addition, the simulations were performed using the SMD under the effect of water, to compare the results with those of the IEFPCM.^14,22^ The comparison between the two models was additionally performed at low dielectric constants of 2.04 and 6.25. All optimizations used tight self-consistent field convergence criteria and an ultrafine integration grid (pruned, 99,590). Vibrational frequency analysis confirmed the absence of imaginary frequencies, verifying that all structures correspond to the true energy minima. Analysis of the electronic wave functions was performed using AIMAll,^23^ which provided the atomic volumes and electron populations required for electrostatic evaluation. The AED of each moiety is defined as the total electron population (∑*N*_*i*_) divided by the total atomic volume (∑*V*_*i*_) of the bioisosteric moiety

$${AED}_{bioisostere}=\frac{\sum N_{i}}{\sum V_{i}}$$

where *N*_*i*_ and *V*_*i*_ are the electron population and volume, respectively, of atom *i*.

## Results and Discussion

### AED across Different Dielectric Constants

Previous studies have shown that bioisosteric moieties tend to exhibit similar AED values, despite differences in their charges, volumes, or populations.^7–15^ This section shows that AED values for bioisosteric moieties also do not correlate with the dipole moments of the corresponding molecules capped with a methyl group.

Figure 2 shows that the net AED of the bioisosteric moieties is insensitive to the various applied dielectric constants, although there will be changes at the atomic level. For example, Figure 3A shows that the volumes of atoms O6 and O9 gradually increase with higher dielectric constants, from 138 to 142 atomic units (a.u.) for O6 and from 137 to 142 a.u. for O9, when ε gradually increases from 0.00 to 78.36. On the other hand, atoms H13, H14, H15, and H16 exhibit slight decreases in their volume with increasing dielectric constant. These opposing trends offset each other, resulting in a nearly constant total volume for the bioisosteric moiety (790 or 791 a.u.), regardless of the dielectric constant. Similarly, the electron populations follow similar trends (but to a lesser extent) with the same atoms. The electron populations of atoms O6 and O9 gradually increase, from 9.06 to 9.13 a.u. for O6 (and even O9), with gradually increasing dielectric constants from 0.00 to 78.36. Therefore, the AED values, calculated as the ratio of electron population to volume, remain largely unaffected by changes in the dielectric constant.

**Figure 2:**
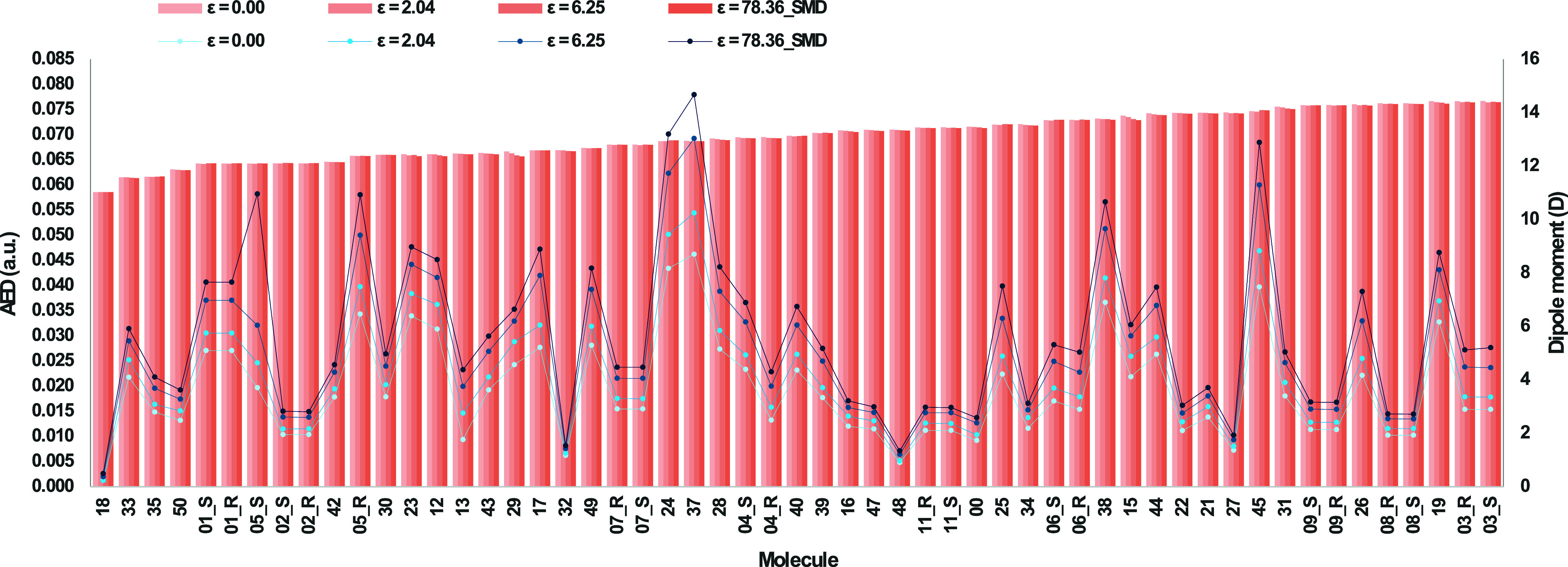
AED (in a.u.), at an isodensity of 0.001 a.u., of carboxylic acid (molecule 00) and all its 63 bioisosteric moieties, and the dipole moment (in Debye, D) for the 64 molecules considered in this study. Both R and S enantiomers of the 11 chiral moieties are considered, thus the annotations “_R” or “_S” for molecules 1 to 11. The AED values and the dipole moments are reported at four different dielectric constants.

**Figure 3:**
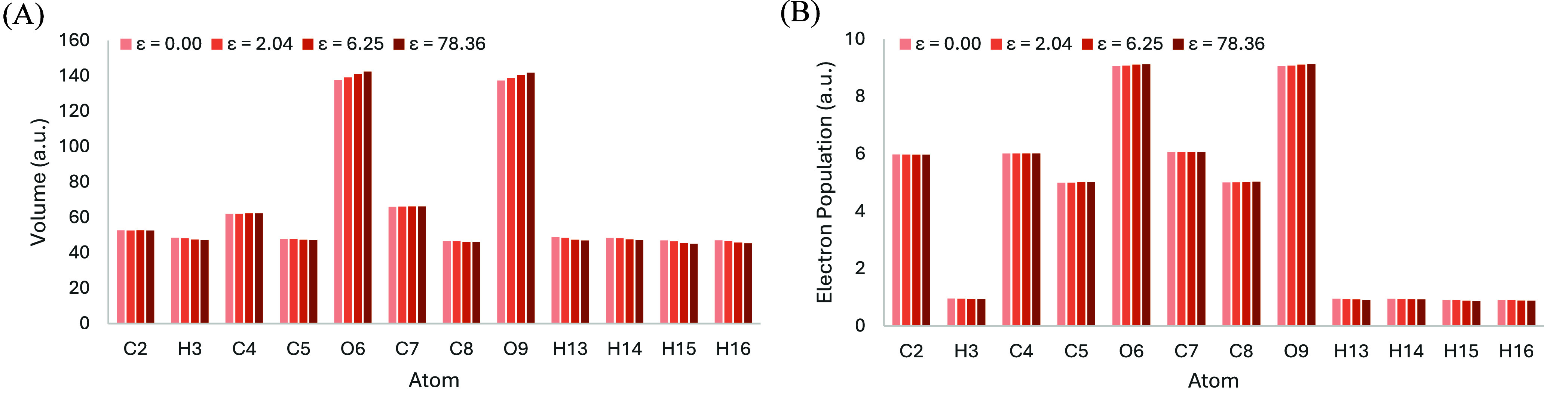
(A) Volumes (in a.u.) and (B) electron populations (in a.u.), at an isodensity of 0.001 a.u., of all the atoms comprising the bioisosteric moiety of molecule 02 (R enantiomer), evaluated under various dielectric constants.

### SMD vs IEFPCM Models

Since SMD and IEFPCM use different parameters such as Coulombic radii, they may perform differently. Therefore, the comparison between the results from the IEFPCM^14^ and SMD models in an aqueous medium (*i.e.* at a dielectric constant of 78.36) was performed. The comparison was additionally performed at low dielectric constants of 2.04 and 6.25.

Figure 4 shows that, although the scales of the dipole moments are off in the SMD vs the IEFPCM solvation models, the trends are similar. However, the AED values do not seem to be sensitive to the solvation method; both models give similar values (see Figure 4) and an R^2^ of 0.99 between the AEDs predicted using the two models (see Figure 5).

**Figure 4:**
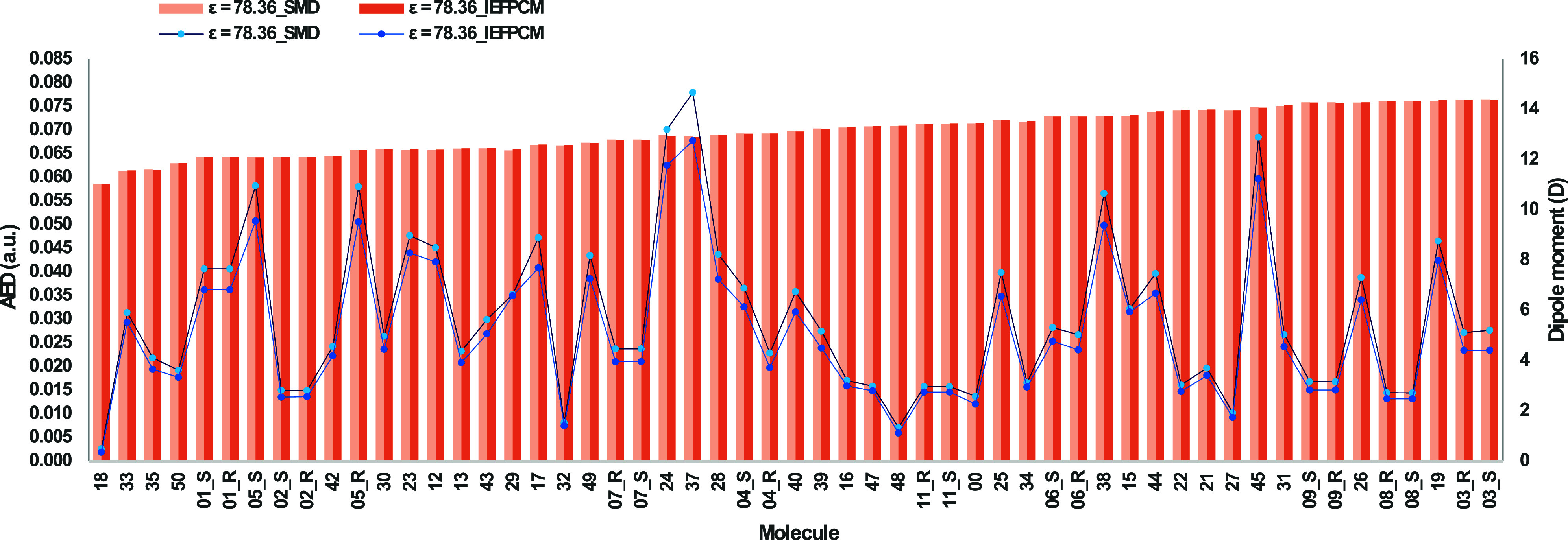
AED (in a.u.), at an isodensity of 0.001 a.u., of carboxylic acid (molecule 00) and its 63 bioisosteric moieties, and the dipole moment (in Debye, D) for the 64 molecules considered in this study. Both R and S enantiomers of the 11 chiral moieties are considered, thus the annotations “_R” or “_S” for molecules 1 to 11. The AED values and the dipole moments are reported at ε = 78.36, using two different solvation models, SMD and IEFPCM.

**Figure 5:**
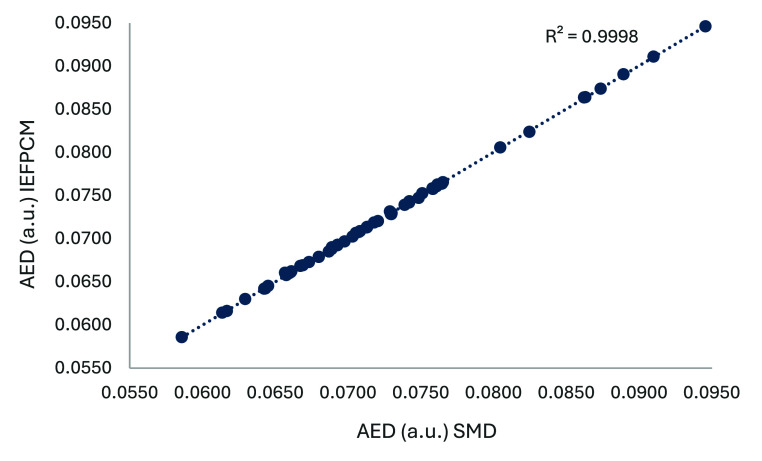
Correlation between AED values (in a.u.) calculated, at an isodensity of 0.001 a.u. and a dielectric constant of 78.36, using the SMD and IEFPCM solvation models. The data points correspond to the 63 carboxylic acid bioisosteric moieties considered in this study.

Although SMD and PCM differ in their theoretical foundations, the AED values obtained from both methods are highly correlated, as shown in Figure 5. Strong correlations between the two methods were consistently observed at dielectric constants of 2.04 and 6.25, each with an R^2^ of 0.99.

## Conclusions

This study shows that the SMD model produces AED values comparable to those in the gas phase, regardless of the dielectric constant chosen to represent different hydrophobic media in biological environments. In addition, the AED tool is equally effective for evaluating bioisosterism using either solvation model (SMD or IEFPCM), whether applied to hydrophobic media or to an aqueous environment. This is useful because it means that both polar and nonpolar media can be equally represented in the context of bioisosteric evaluations using the AED tool. These findings suggest that the AED tool can be reliably used in the lead optimization of candidate molecules under various biologically relevant conditions.
